# An integrated environmental improvement of marshlands: impact on control and elimination of schistosomiasis in marshland regions along the Yangtze River, China

**DOI:** 10.1186/s40249-017-0287-1

**Published:** 2017-03-22

**Authors:** Le-Ping Sun, Wei Wang, Yin-Ping Zuo, Zheng-Qiu Zhang, Qing-Biao Hong, Guo-Jing Yang, Hong-Ru Zhu, You-Sheng Liang, Hai-Tao Yang

**Affiliations:** 1 Key Laboratory of National Health and Family Planning Commission on Parasitic Disease Control and Prevention, No. 117 Yangxiang, Meiyuan, Wuxi City, Jiangsu Province 214064 China; 2 Jiangsu Provincial Key Laboratory on Parasites and Vector Control Technology, No. 117 Yangxiang, Meiyuan, Wuxi City, Jiangsu Province 214064 China; 3 Jiangsu Institute of Parasitic Diseases, No. 117 Yangxiang, Meiyuan, Wuxi City, Jiangsu Province 214064 China; 4 Yangzhou Municipal Center for Disease Control and Prevention, No. 36 Yanfu East Road, Yangzhou City, Jiangsu Province 225000 China; 5 Yizheng County Center for Disease Control and Prevention, NO. 1 Jiankang Road, Yangzhou City, Jiangsu Province 211440 China

**Keywords:** Schistosomiasis, *Oncomelania hupensis*, Environmental improvement, Marshland regions, Yangtze River basin, China

## Abstract

**Background:**

Schistosomiasis is a global snail-transmitted infectious disease of poverty. Transmission control had been achieved in China in 2015 after the control efforts for over 60 years. Currently, the remaining core regions endemic for *Schistosoma japonicum* are mainly located in the marshland and lake regions along the Yangtze River basin.

**Methods:**

During the period from 2001 through 2015, an integrated environmental improvement of the marshlands was carried out through the implementation of industrial, agricultural and resources development projects in Yizheng County along the Yangtze River. *S. japonicum* infection in humans, livestock and snails was estimated by serology, stool examination, hatching technique and microscopy during the 15-year study period to evaluate the effect of the integrated environmental improvement on control and elimination of schistosomiasis.

**Results:**

A 0.05% overall rate of *S. japonicum* infection was observed in snails during the 15-year study period, and no infected snails were detected since 2012. The overall prevalence of *S. japonicum* infection was 0.09% in humans during the study period, and no human infection was found since 2012. In addition, only 13 bovines were identified with *S. japonicum* infection in 2003 during the 15-year study period, and since 2004, no infection was found in livestock.

**Conclusion:**

The results of the present study demonstrate that the implementation of industrial, agricultural and water resources development projects, not only alters snail habitats in marshland regions, and promotes local economic development, which appears a win-to-win strategy to block the transmission of *S. japonicum* and accelerate socio-economic development along the Yangtze River.

**Electronic supplementary material:**

The online version of this article (doi:10.1186/s40249-017-0287-1) contains supplementary material, which is available to authorized users.

## Multilingual abstracts

Please see Additional file 1 for translations of the abstract into the six official working languages of the United Nations.

## Background

Schistosomiasis, caused by the blood fluke of the genus *Schistosoma*, is a snail-transmitted infectious disease of poverty that ranks second only to malaria in terms of morbidity and mortality among tropical parasitic diseases [1–3]. In China, schistosomiasis was once endemic in 453 counties of 12 provinces south of the Yangtze River [4–6]. According to the environmental ecosystems and snail habitats, schistosomiasis-endemic regions are classified into three types in China, including marshland and lake regions, plain regions with waterway network, and hilly and mountainous regions [7], and a “infection control-transmission control-transmission interruption-elimination” four-stage roadmap has been developed [8–10]. Since the initiation of the national schistosomiasis control program at early 1950s [11–13], schistosomiasis control has been given a high priority in China [14, 15], and multiple integrated strategies have been proposed according to the epidemiological profile and intensity of transmission, which have resulted in a great success in schistosomiasis control in the country [16]. Notably, an integrated strategy with emphasis on infectious source control was developed in 2004 [17], and the wide implementation of the strategy results in a huge reduction in *S. japonicum* infection in humans, livestock and snails in China [18–32]. By 2015, transmission control of schistosomiasis has been achieved across the country [33], indicating less than 1% *S. japonicum* infection in both humans and livestock, no local acute infections, and no infected snails for successive 2 years throughout the country [34–36].

Currently, the remaining core regions endemic for *S. japonicum* are mainly located in the marshland and lake regions along the Yangtze River basin [37]. *Oncomelania hupensis*, the intermediate host of *S. japonicum*, is widely distributed in the shores of rivers and lakes along the middle and lower reaches of the Yangtze River [38], and the marshland is characterized by “land in winter, water in summer” due to seasonal tide, which provides an ideal environment for snail growth and reproduction [39–41]. In addition, there are a huge number of livestock [42, 43] and a variety of wild animals on the marshland [44], and plenty of boatmen and fishermen living along the marshlands frequently contact with *S. japonicum*-infested water [45–47], which complicate the control efforts, and result in frequent resurgence of schistosomiasis in the marshland regions of China [40]. Completely blocking the transmission of *S. japonicum* in the marshland regions is therefore critical to and it has become the primary task for elimination of schistosomiasis in China [48].

In order to effectively control the transmission of *S. japonicum* in the marshland regions, multiple integrated interventions have been developed, which are based on inter-sectoral collaboration between governmental departments from agriculture, health, water resource development, forestry, and land and resources [49]. The optimization and combination of these integrated strategies has been proved to greatly facilitate the progress towards the elimination of schistosomiasis in China [50–52]. Previous studies have demonstrated that a thorough and integrated modification of the snail habitats is required in order to completely block the transmission of *S. japonicum* in the marshland regions [53–55].

Since 2001, industrial development was initiated in Yizheng County along the Yangtze River, and an integrated environmental improvement was simultaneously performed targeting the marshland outside the embankment. In the current study, we evaluated the effect of this integrated environmental improvement of the marshlands on control and elimination of schistosomiasis in marshland regions of Yizheng County along the Yangtze River, China, from 2001 to 2015.

## Methods

### Study site

Yizheng County is located on the north bank of the lower reaches of the Yangtze River basin, which covers an area of 901 km^2^, and has a population of 600 thousand. Currently, there are 5 out of 12 townships and 31 out of 149 villages endemic for *S. japonicum*, with 260 thousand people at risk of infection. There are 12.6 thousand accumulated schistosomiasis cases, 1.79 thousand accumulated infected bovines, and accumulated snail habitats of about 9.14 km^2^ detected in Yizheng County.

### Integrated environmental improvements of marshlands

Based on the epidemiological profile of the marshland regions, an integrated environmental improvement of the marshlands were carried out in Yizheng County through the implementation of industrial development projects from 2001 to 2004, and agricultural and water resources development projects conducted between 2005 and 2011. Three projects were implemented for industrial development. (1) Building boat factories. Firstly, all wild trees and grasses were cleaned on the marshland, and the snail habitats were given molluscicide treatment. Then, the soil containing snails was deeply buried with river sand, and the marshland was made flat. Finally, boat factories were built on the marshland, and tap-water treatment systems and public latrines with three-cell septic tanks were constructed. (2) Building docks and harbors. The wild trees and grasses were cleaned on the marshland, and docks and harbors were built with concrete. (3) Building ecological parks. The marshland was subjected to environmental improvement, and ornamental plants were grown in high-elevation marshlands, while fish was raised in low-elevation marshlands to ensure no snail survival in the ecological parks.

Agricultural development projects consisted of land reclamation and planting, aquatic product breeding, and building trees. (1) Land reclamation and planting. Wild trees and reeds were cleaned from high-elevation marshlands, which were then subjected to mechanized plough and molluscicide treatment. Economic crops were planted each year during the non-flood period, such as wheat and rape. (2) Aquatic product breeding. Fish ponds were constructed in low-elevation marshlands to breed aquatic products, such as fish and shrimp. (3) Building trees. *Populus nigra* was grown in marshlands that were not suitable for land reclamation and planting, and aquatic product breeding after reeds were removed, to achieve environmental improvement of the snail habitats.

Water resources development projects consisted of hardening river banks with concrete, building sluices for prevention of snail spread and construction of snail-retention pools to allow no snail entry into ditches.

The gross national product (GNP) and output values due to industrial, agricultural and water resources development were collected in Yizheng County during the study period from 2001 through 2015, and the proportion of the output values of economic development in GNP was estimated each year.

### Effect of the integrated environmental improvements on schistosomiasis elimination

During the period from 2001 to 2015, the study villages were selected from Yizheng County using the clustering sampling method [56]. Briefly, the 31 endemic villages were classified into three types (low, moderate and high) according to the endemicity, and two villages were randomly selected from each type. All residents living in the selected villages were identified for *S. japonicum* infection by using serological screening followed by stool examinations. The individuals were detected for specific IgG antibodies against *S. japonicum* with a dipstick dye immunoassay (DDIA) during the schistosomiasis non-transmission period [57–59], and then all seropositives received a miracidium hatching test (3 individual hatches read blind, of 50 grams faces per hatching) [60]. The annual rate of human *S. japonicum* infection was estimated using the following formula: rate of human *S. japonicum* infection (%) = No. of egg-positives/No. of residents examined × 100%. Acute schistosomiasis was diagnosed based on the following criteria: (1) A history of contact with *S. japonicum*-infested water during the past 2 weeks to 3 months; (2) presence of fever, hepatomegaly and peripheral blood eosinophilia, complicated by splenomegaly, cough, liver tenderness, abdominal distension or diarrhea; and (3) detection of *S. japonicu*m egg or miracidium [61].

At spring and autumn of each year, all livestock were detected for *S. japonicum* infection with a miracidium hatching test [62], and the prevalence of *S. japonicum* infection was calculated in livestock.

At spring each year, snail survey was performed in ditches inside the embankment and in marshlands outside the embankment using a systematic method [38]. A snail collection device, measuring 0.1 m^2^, was placed every 5 (in ditches) or 15 m (in marshlands) along the survey line. All snails within the device were captured, transferred to laboratory and identified for *S. japonicum* infection under a microscope. The area of snail habitats and infected snail habitats was recorded, and the rate of *S. japonicum* infection in snails was estimated.

### Ethical consideration

This study was approved by the Ethical Review Committee of Jiangsu Institute of Parasitic Diseases (permission number: IRB00001037). Animal experiments were done following the Guidelines for the Care and Use of Laboratory Animals, and signed informed consent was obtained from all subjects participating in the study.

### Data management and analysis

All data were processed and analyzed in Microsoft Excel version 2007 (Microsoft Corporation; Redmond, WA, USA).

## Results

### Implementation of integrated environmental improvements of marshlands

**Fig. 1:**
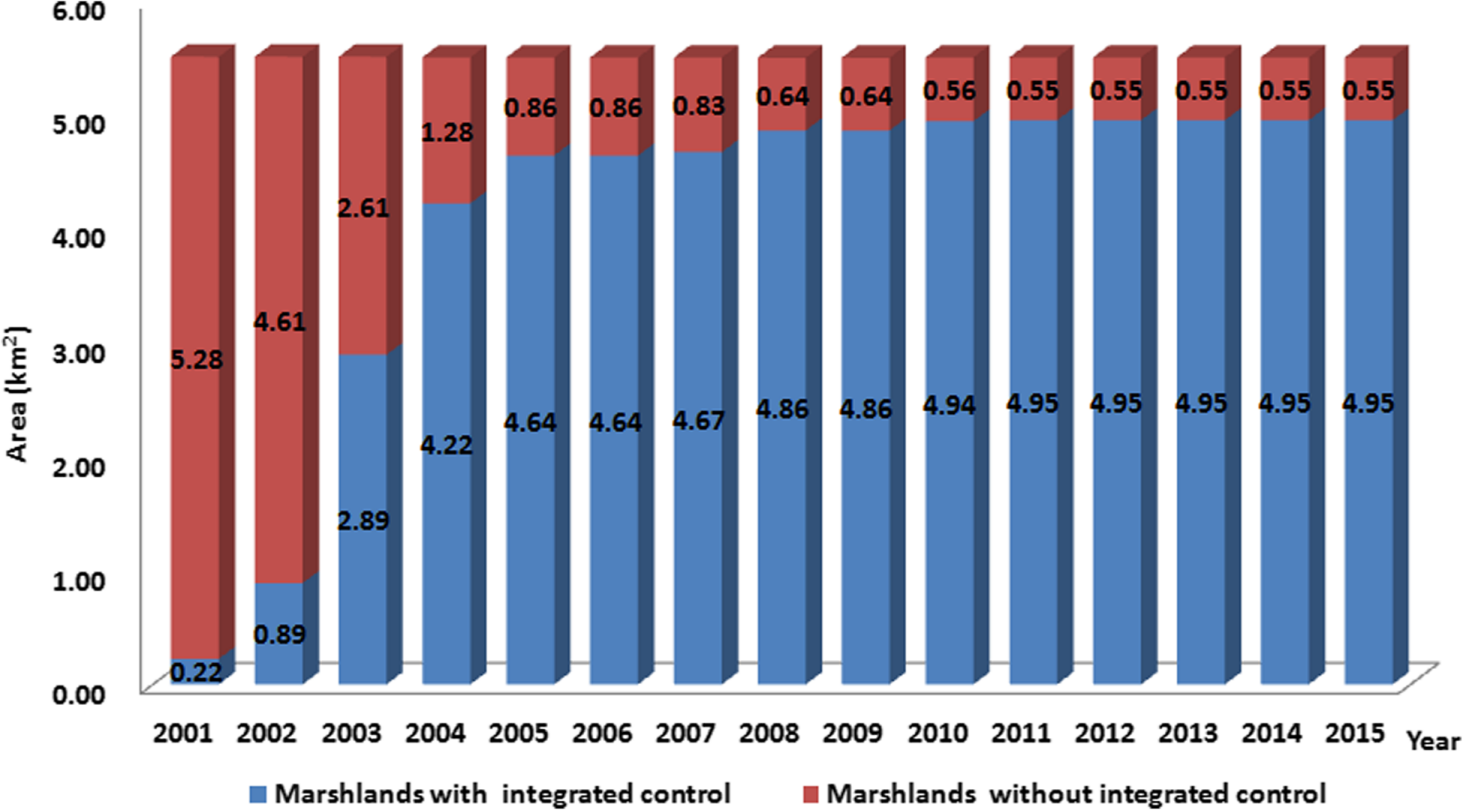
Annual area of the marshlands with and without integrated environmental improvements in Yizheng County along the Yangtze River from 2001 to 2015

**Fig. 2:**
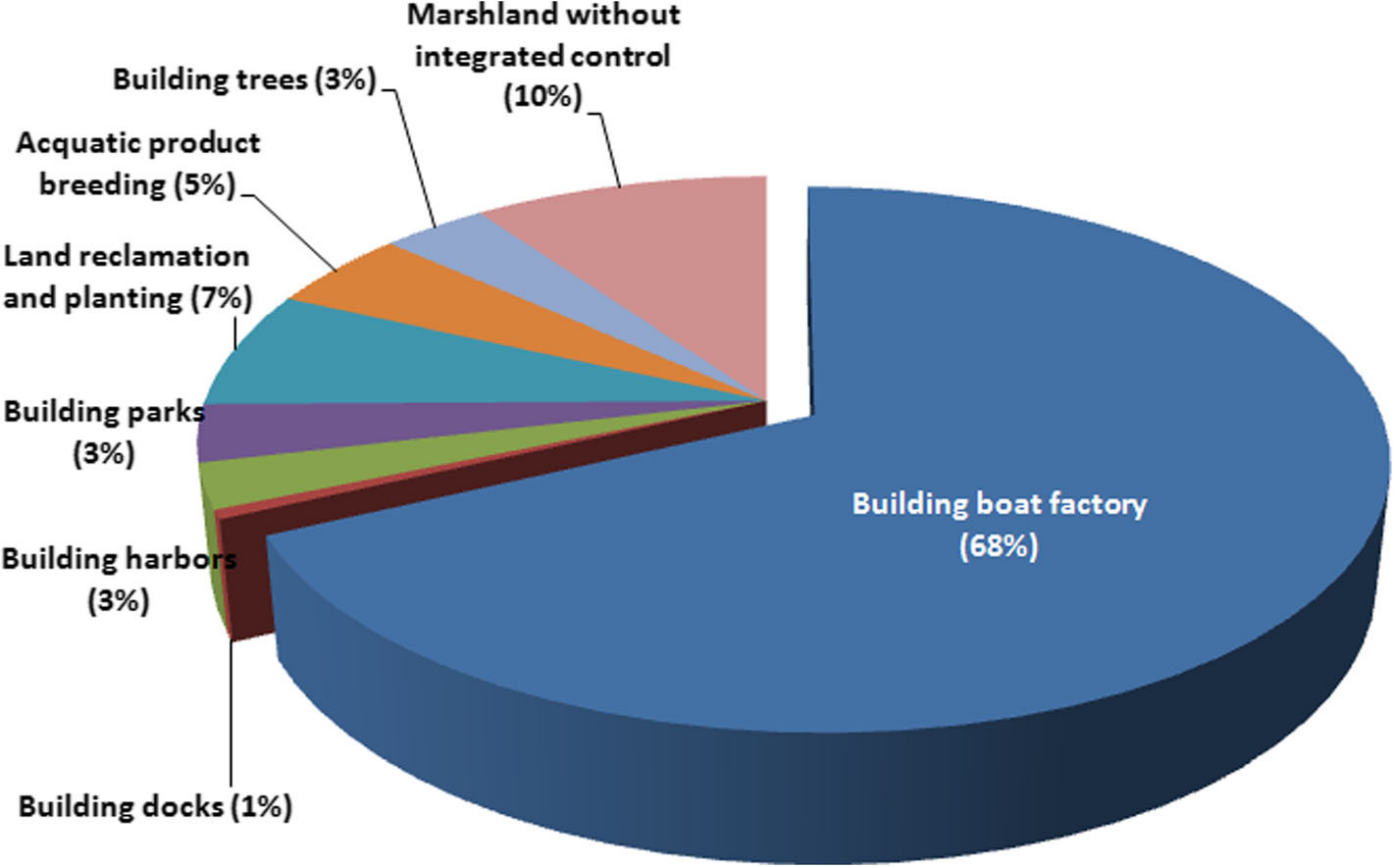
Constitution of interventions implemented for the integrated environmental improvements of marshlands in Yizheng County along the Yangtze River from 2001 to 2015

During the period from 2001 through 2015, integrated environmental improvements were implemented in marshlands outside the embankment of Yizheng County, with a total area of 4.95 km^2^. The rate of environmental improvement appeared a rise over the study period, and was 90% in 2015 (Table 1, Fig. 1). By 2015, there were only 6 marshlands covering an area of 0.55 km^2^ that remained to be improved, consisting of 10% of the total marshlands outside the embankment (Fig. 2).

**Table 1:** Integrated improvements of marshlands in Yizheng County along the Yangtze River during the period from 2001 through 2015

Year	Status of integrated control				Rate of marshlands with integrated improvements in all marshlands (%)
	Interventions	Integrated improvements of marshlands at local year (km^2^)	Marshlands with integrated improvements (km^2^)	Marshlands without integrated improvements (km^2^)	
2001	Building factory and trees	0.22	0.22	5.28	4
2002	Building factory and burying snails with river sand	0.67	0.89	4.61	16.18
2003	Building factory and burying snails with river sand	2	2.89	2.61	52.55
2004	Building factory and burying snails with river sand	1.33	4.22	1.28	76.73
2005	Building fish ponds	0.42	4.64	0.86	84.36
2006	None	0	4.64	0.86	8436
2007	Building docks	0.03	4.67	0.83	84.91
2008	Building ecological parks	0.19	4.86	0.64	88.36
2009	None	0	4.86	0.64	88.36
2010	Plough and planting	0.08	4.94	0.56	89.82
2011	Building docks	0.01	4.95	0.55	90
2012	None	0	4.95	0.55	90
2013	None	0	4.95	0.55	90
2014	None	0	4.95	0.55	90
2015	None	0	4.95	0.55	90

During the period from 2001 to 2015, a total of 15 marshlands outside the embankment were subjected to industrial development, which covered an area of 4.12 km^2^ and consisted of 74.91% of the total marshlands, and there were 3.75 (68.18%), 0.03 (0.55%), 0.15 (2.73%) and 0.19 km^2^ marshlands (3.45%) used for building boat factories, docks, harbors and ecological parks, respectively (Fig. 2). Agricultural development was conducted in 4 marshlands, which covered an area of 0.84 km^2^ and consisted of 15.27% of the total marshlands, and there were 0.39 (7.09%), 0.26 (4.73%) and 0.19 km^2^ marshlands (3.45%) with land reclamation and planting, aquatic product breeding, and trees building, respectively (Table 1, Fig. 2). In addition, 6 river banks were hardened with concrete with 4.87 km in length, and 6 sluices/snail-retention pools were built.

During the 15-year study period, the total output values due to industrial, agricultural and water resources development consisted of 44.23% of the total GNP in Yizheng Country from 2001 through 2015, and the rate of the output values of economic development in the total GNP appeared a tendency towards a rise between 2001 and 2012 (Table 2).

**Table 2:** Proportion of the output values of economic development in GNP in Yizheng County from 2001 to 2015

Year	GNP in Yizheng County (Billion Yuan)	Output values of industrial, agricultural and water resources development (Billion Yuan)	Proportion of the output values of economic development in GNP (%)
2001	5.67	1.86	32.8
2002	6.11	2.2	36.01
2003	7.17	3.35	46.72
2004	8.81	4.73	53.69
2005	10.6	6.13	57.83
2006	13.01	7.52	57.8
2007	16.25	8.98	55.26
2008	20.06	9.12	45.46
2009	22.73	12.3	54.11
2010	27.82	16.7	60.03
2011	33.35	24.3	72.86
2012	37.02	25.5	68.88
2013	41.02	11.16	27.21
2014	45.45	10.15	22.33
2015	50.2	8.71	17.35
Total	345.27	152.71	44.23

### *S. japonicum* infection in snails

**Fig. 3:**
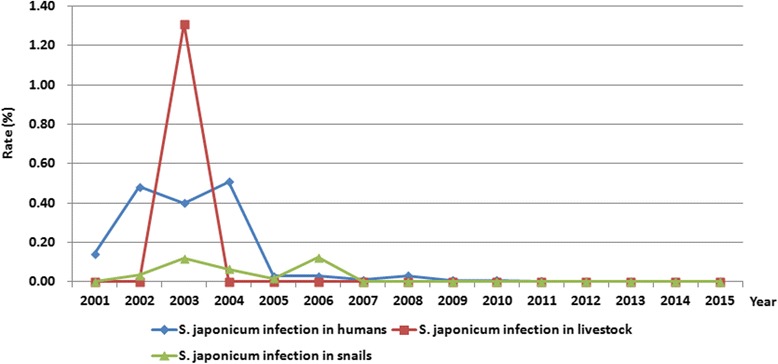
Annual rate of *S. japonicum* infection in humans, livestock and snails in Yizheng County along the Yangtze River from 2001 to 2015

During the period from 2001 to 2015, a total of 72 095 snails were captured and examined for *S. japonicum* infection, and 34 snails were identified with infection, with a 0.05% overall infection rate observed. Since 2007, no infected snails were detected (Fig. 3). In addition, a total of 11.09 km^2^ snail habitats were identified during the 15-year study period. During the first 7 years of the integrated control, snail habitats were rapidly shrunk, and the area of snail habitats reduced by 80.47% in 2007 relative to 2001, with an annual decline of 13.41%. Since 2012, no snails were found. A total of 0.52 km^2^ infected snail habitats were identified from 2001 to 2015, and 98.08% were detected between 2002 and 2005. Since 2007, no infected snail habitats were found (Table 3).

**Table 3:** Results of snail survey in Yizheng County along the Yangtze River during the period from 2001 through 2015

Year	Snail survey (km^2^)	Area of snail habitats (km^2^)		*S. japonicum* infection in snails		Area of infected snail habitats (km^2^)
		Outside embankment	Inside embankment	No. snails examined	Infection in snails (%)	
2001	16.8	1.87	0.05	9464	0	0
2002	16.8	1.89	0.06	11955	0.03	0.02
2003	16.8	1.79	0.1	14175	0.12	0.16
2004	16.82	1.59	0.13	12177	0.07	0.24
2005	16.82	1.59	0.13	11938	0.02	0.09
2006	16.82	0.72	0.14	2445	0.12	0.01
2007	16.82	0.37	0.005	4342	0	0
2008	16.82	0.17	0.005	849	0	0
2009	9.14	0.14	0.009	1111	0	0
2010	9.14	0.15	0.008	2287	0	0
2011	9.14	0.16	0.009	1352	0	0
2012	9.14	0	0	0	0	0
2013	9.14	0	0	0	0	0
2014	9.14	0	0	0	0	0
2015	9.14	0	0	0	0	0

### *S. japonicum* infection in humans and livestock

**Fig. 4:**
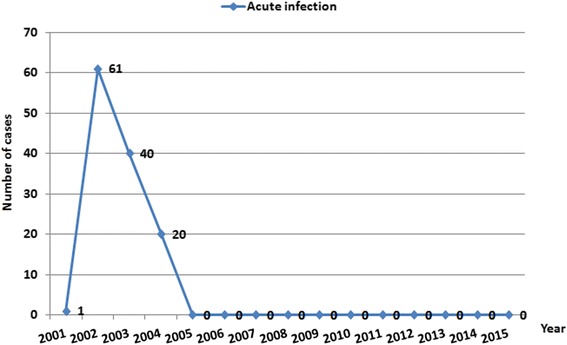
Annual number of acute human *S. japonicum* infection in Yizheng County along the Yangtze River from 2001 to 2015

From 2001 to 2015, a total of 316 290 residents were detected for *S. japonicum* infection, and 276 individuals were identified with infections, with a 0.09% overall prevalence of *S. japonicum* infection. Most of the human *S. japonicum* infection was detected in the first 4 years of the integrated control between 2001 and 2004, which consisted of 88.77% of the total infections. No human infection was found since 2012 (Fig. 3). In addition, there were 122 acute infections identified during the 15-year study period, and all cases were detected between 2001 and 2004. Since 2005, no acute infection was observed (Fig. 4).

During the 15-year study period, a total of 15583 livestock were detected for *S. japonicum* infection, and only 13 bovines were identified with infection in 2003. Since 2004, no *S. japonicum* infection was found in livestock (Fig. 3).

Following 5-year integrated environmental improvements of the marshlands, infection control was achieved in Yizheng County in 2005, and transmission control was achieved in 2007. In addition, the transmission of *S. japonicum* has been interrupted in the county since 2013.

## Discussion

Previous studies have demonstrated that elimination of the source of *S. japonicum* infection or the intermediate host snail is the key intervention leading to interruption of schistosomiasis transmission [63]. However, the source of *S. japonicum* infection is difficult to be eliminated in marshland regions since (1) A variety of wild animals, which may serve as reservoir hosts for *S. japonicum*, are living in marshlands, which complicate the control efforts [44]; (2) The massive marshlands are ideal sites for the gathering of livestock [42]; and (3) A large number of mobile boatmen and fishermen are living and working along the Yangtze River, which increases the risk of importation of infectious sources [45–47]. In addition, the extensive snail dispersal due to flooding, and the high reproduction rate of residual snails result in a low possibility for elimination of snails from the marshland regions [39].

In this study, the integrated environmental improvements of the marshland regions experienced a long period of time, which may be classified into early- (environmental modification), intermediate- (building factory), and late-stages (surveillance). At the early stage, a large number of workers lived and worked in the marshlands which had a high risk of *S. japonicum*, and acute infection easily occurred. Our data showed a total of 122 acute cases during the period between 2001 and 2004, suggesting that individual protection is required for workers on the marshlands. At the intermediate stage, workers were busy in building factories, and had a reduction in the frequency to contact with infested water, resulting in a decline in human *S. japonicum* infection. Therefore, management of human feces and provision of access to safe water is required at this stage. At the late stage, the monitoring of *S. japonicum* infection in newly recruited workers should be strengthened to consolidate the control achievements, since no local source of *S. japonicum* infection was detected. Following 15-year integrated control, there were 10% of the total marshland that remained to be developed, and reeds are predominant vegetations on these marshlands, which are potential snail habitats and potential threat to resurgence of schistosomiasis. The surveillance and control of snails on the remaining marshlands without integrated improvements is required to consolidate the control efforts. In addition, the monitoring of external sources of *S. japonicum* infection should be strengthened to prevent the importation of mobile sources of *S. japonicum* infection from the Yangtze River, so as to achieve the elimination of schistosomiasis.

During the 15-year study period from 2001 through 2015, the total output values due to economic development consisted of 44.23% of the total GNP in the study site, and the rate of the output values of economic development in the total GNP showed an increasing tendency between 2001 and 2012. Our data demonstrate that the industrial, agricultural and water resources development projects facilitate local economic development in Yizheng County, and achieve complete improvements of snail habitats on the marshlands. The integrated environmental improvements of marshlands resulted in a rapid transfer from a hyper-endemic region to transmission interruption of schistosomiasis, and elimination of this infectious disease of poverty as a public health concern in Yizheng County. This strategy may provide new insights into the elimination of schistosomiasis in developing countries.

Our study has a limitation that no parallel control was designed. During the study period, the *National Mid- and Long-term Plan for the Prevention and Control of Schistosomiasis in China (2004–2015)* was implemented in the study site [17], which increased the difficulty to assign a parallel control. However, our data indicated the integrated environmental improvements of marshlands played a leading role in the control and elimination of schistosomiasis in Yizheng County. In addition, the Three Gorges Dam was operated during the study period [64], which may positively or negatively affect the progress towards the elimination of schistosomiasis in the middle and lower reaches of the Yangtze River [65]. The changes of water levels caused by the Three Gorges Dam operation result in the extension of the duration of marshland exposure, which may increase the humans and livestock activities on marshlands. This may increase the risk of schistosomiasis transmission. However, the effective regulation of the water levels by the Dam may reduce the anti-flood pressure in the lower reaches of the Yangtze River, and previously non-utilized marshlands may be gradually developed. Our findings also showed that the integrated environmental improvements of marshlands achieved double benefits of promoting local economic development and eliminating schistosomiasis.

## Conclusions

The results of the present study demonstrate that the integrated environmental improvement of marshlands through the implementation of industrial, agricultural and water resources development projects, not only alters snail habitats in marshland regions, and promotes local economic development, which appears a win-to-win strategy to block the transmission of *S. japonicum* and accelerate socio-economic development along the Yangtze River. Our findings provide new insights into the development of strategies for transmission interruption and elimination of schistosomiasis in developing countries.

## Additional file

Additional file 1: Multilingual abstracts in the six official working languages of the United Nations. (PDF 688 kb)

## Abbreviations

DDIA: Dipstick dye immunoassay
GNP: Gross national product
