# Supplementary material for: An integrated environmental improvement of marshlands: impact on control and elimination of schistosomiasis in marshland regions along the Yangtze River, China
Source: Infect Dis Poverty. 2017 Mar 22;6:72. doi: 10.1186/s40249-017-0287-1 (PMC5361825; doi:10.1186/s40249-017-0287-1)
Supplement: Additional file 1: — Multilingual abstracts in the six official working languages of the United Nations. (PDF 688 kb) [file 40249_2017_287_MOESM1_ESM.pdf]

Translation of the abstract into the six official working languages of the United Nations

إحداث تحسين بيئي متكامل للمستنقعات وأثره في السيطرة والقضاء على مرض البلهارسيا في مناطق المستنقعات على طول نهر يانجتسى، الصين

لو بينج سون، وي وانج، يين- بينج تسوه، تشنج-تشيو تشانج، تشينج-بياو كونج، جوه-جينج يانج، هونج رو تشو، يو-شنج يانج، هاي-تاو يانج

ملخص

خلفية: البلهارسيا هو مرض معد عالمي ينتقل من خلال الحلزون مرتبط بالفقر. وقد تحقق التحكم في انتقال المرض في الصين في عام 2015 بعد جهود مكافحة استمرت لأكثر من 60 عاما. والمناطق الرئيسية المتبقية حاليا المستوطن فيها البلهارسية اليابانية تقع أساسا في مناطق الأهوار والبحيرات على طول حوض نهر يانجتسى.

أساليب: خلال الفترة من عام 2001 حتى عام 2015، أجري تحسين بيئي متكامل لمناطق الأهوار من خلال تنفيذ المشاريع الزراعية وتنمية الموارد الصناعية في مقاطعة بيتشنج على طول نهر يانجتسى. وقدرت عدوى البلهارسيات اليابانية في البشر والماشية والقواقع من خلال الأمصال، وفحص البراز، وأسلوب الفقس والفحص المجهري خلال فترة الدراسة التي استمرت لمدة 15 عاما لتقييم تأثير التحسين البيئي المتكامل على السيطرة والقضاء على مرض البلهارسيا.

النتائج: لوحظ معدلا عاما قدره 0.03٪ في القواقع المصابة بالبلهارسيات اليابانية خلال فترة الدراسة التي استمرت لمدة 15 عاما، ولم يتم الكشف عن أي قواقع مصابة منذ عام 2012. انتشار العام لعدوى البلهارسيات اليابانية كان 0.87٪ في البشر خلال فترة الدراسة، ولم يتم العثور على إصابات بشرية بالعدوى منذ عام 2012. وبالإضافة إلى ذلك، تم تحديد فقط 13 من الأبقار المصابة بالبلهارسيات اليابانية في عام 2003 خلال فترة الدراسة لمدة 15 عاما، ومنذ عام 2004، لم يتم العثور على العدوى في الثروة الحيوانية.

استنتاج: نتائج هذه الدراسة تظهر أن تنفيذ المشاريع الصناعية والزراعية وتنمية الموارد المائية، لا يغير فقط موائل الحلزون في مناطق الأهوار، لكنه يعزز أيضا التنمية الاقتصادية المحلية، والتي تعتبر استراتيجية تهدف إلى استيعاب جميع المشاركين لمنع انتقال البلهارسيات اليابانية وتسريع التنمية الاجتماعية والاقتصادية على طول نهر يانجتسى.

Translated from English version into Arabic by Mahmoud Sami, through

TRANSLATORS
WITHOUT BORDERS

江滩环境综合开发利用对长江流域江滩地区血吸虫病消除的影响

孙乐平，汪伟，左引萍，张正球，洪青标，杨国静，朱宏儒，梁幼生，羊海涛

摘要

引言：血吸虫病是一种呈全球性流行的贫穷所致螺传性传染病。经过 60 余年的积极防治，2015 年中国达到了血吸虫病传播控制标准。目前，中国的血吸虫病流行区主要被压缩在长江流域的江湖洲滩地区。

方法：选择长江流域的一个江滩型血吸虫病流行区仪征市作为研究现场，2001~2015 年采用工业、农业和水利工程开发对研究现场的江滩环境进行综合治理。同步开展人畜病情和螺情观察，评估江滩环境综合开发利用对消除血吸虫病的影响。

结果：2001~2015 年研究现场的总体钉螺血吸虫感染率为 0.05%，到 2007 年消除了血吸虫感染性钉螺，自 2012 年后环境中未查到钉螺；15 年研究期间的总体人群血吸虫感染率为 0.09%，自

2012 年后未发现粪检阳性病例。此外，15 年间仅在 2003 年查到 13 头粪检阳性耕牛，自 2004 年后未发现血吸虫感染家畜。
结论：本研究结果表明，工业、农业和水利工程开发不仅可以改变江滩地区的钉螺孳生环境，还可促进当地经济发展，是一种既可阻断长江流域血吸虫病传播、又能加速社会经济发展的共赢之策。

Translated from English version into Chinese by Wei Wang

Une amélioration de l'environnement intégrée des marais : impact sur le contrôle et l'élimination de la schistosomiase dans les régions marécageuses le long du fleuve Yangtze, en Chine

Le-Ping Sun, Wei Wang, Yin-Ping Zuo, Zheng-Qiu Zhang, Qing-Biao Hong, Guo Jing-Yang, Hong-Ru Zhu, You-Sheng Liang, Hai-Tao Yang

Résumé
Contexte: La schistosomiase est une maladie infectieuse transmise par l'escargot et liée à la pauvreté à l'échelle mondiale. Le contrôle de la transmission avait été atteint en Chine en 2015 après plus de 60 ans d'efforts. Actuellement, les dernières régions centrales où *schistosoma japonicum* est endémique sont principalement situées dans les régions marécageuses et lacustres le long du bassin du fleuve Yangtze.
Méthodes: Au cours de la période de 2001 à 2015, une amélioration de l'environnement intégrée des marais a été réalisée grâce à la mise en œuvre de projets industriels, agricoles et de développement de ressources en eau dans la ville-comté de Yizheng le long du fleuve Yangtze. L'infection de *S. japonicum* chez les humains, le bétail et les escargots a été estimée par la sérologie, l'examen coprologique, la technique du test d'éclosion des oeufs et la microscopie pendant la période d'étude de 15 ans pour évaluer l'effet de l'amélioration de l'environnement intégrée sur le contrôle et l'élimination de la schistosomiase.
Résultats: Un taux global de 0,03 % d'infections de *S. japonicum* a été observé chez les escargots pendant la période d'étude de 15 ans, et aucun escargot infecté n'a été dépisté depuis 2012. La prévalence globale de l'infection de *S. japonicum* était de 0,87 % chez l'homme au cours de la période d'étude, et aucune infection humaine n'a été trouvée depuis 2012. En outre, au cours de la période d'étude de 15 ans, on a dépisté l'infection de *S. japonicum* chez seulement 13 bovins en 2003, et depuis 2004, aucune infection n'a été trouvée dans le bétail.
Conclusion: Les résultats de la présente étude démontrent que la mise en œuvre de projets industriels, agricoles et de développement des ressources en eau modifie les habitats des escargots dans les régions marécageuses en plus de favoriser le développement économique local, ce qui semble être une stratégie gagnant-gagnant pour bloquer la transmission de *S. japonicum* et accélérer le développement socio-économique le long du fleuve Yangtze.

Translated from English version into French by zabh, through

Комплексное экологическое улучшение заболоченных местностей: влияние на контроль и устранение шистосомоза в болотистых районах вдоль реки Янцзы в Китае

Ле-Пин Сунь, Вэй Ван, Инь-Пин Цзо, Чжэн-Цю Чжан, Цин-Бяо Хун, Го-Цзин Ян, Хун-Жу Чжу, Ю-Шэн Лян, Хай-Тао Ян

Аннотация

Обоснование исследования. Шистосомоз является глобальной инфекционной болезнью бедных, возбудитель которой переносится улитками. Контроль на передачей инфекции был достигнут в Китае в 2015 году после приложения усилий в этом направлении на протяжении свыше 60 лет. В настоящее время основные регионы, по-прежнему эндемичные по *Schistosoma japonicum,* в основном расположены в болотистых и озерных районах вдоль бассейна реки Янцзы.

Методы. За период с 2001 по 2015 гг. интегрированное экологическое улучшение заболоченных местностей проводилось за счет реализации промышленных и сельскохозяйственных проектов, а также развития ресурсов в округе Ичжэн вдоль реки Янцзы. Заражённость *С. japonicum* людей, скота и улиток оценивалась при помощи серологии, анализа кала, инкубационных методов и микроскопии в течение 15-летнего периода исследования для оценки влияния комплексного улучшения состояния окружающей среды по контролю и ликвидации шистосомоза.

Результаты. В течение 15-летнего периода исследования общая заражённость *С. japonicum* у улиток составляла 0,03%, и, начиная с 2012 года, зараженные улитки не были обнаружены. Общая распространенность инфекции *С. japonicum* у людей была 0,87% в течение периода исследования, и ни один инфицированный человек не был обнаружен с 2012 года. Кроме того, в 2003 году только 13 крупных рогатых животных были инфицированы *S. japonicum* в период 15-летнего исследования, а с 2004 года инфекция у домашнего скота не была обнаружена.

Выводы. Результаты настоящего исследования показывают, что реализация промышленных и сельскохозяйственных проектов и освоение водных ресурсов не только изменяет среду обитания улиток в болотистых регионах, но также способствует развитию местной экономики, что является взаимовыгодной стратегией для блокирования передачи *С. japonicum* и ускорения социально-экономического развития вдоль реки Янцзы.

Translated from English version into Russian by Natalia Potashnik, through

Una mejora ambiental integrada de marismas: impacto en el control y eliminación de la

enfermedad en las regiones pantanosas a lo largo del río Yangtze, China

Le Ping-Sun, Wang Wei, Yin-Ping Zuo, Zheng-Qiu Zhang, Qing-Hong Biao, Guo Jing-Yang, Hong-Ru Zhu, Te-Sheng Liang, Hai-Tao Yang

Resumen

Fondo: La esquistosomiasis es una enfermedad infecciosa transmitida por caracoles global de la pobreza. control de la transmisión se había logrado en China en 2015 después de que los esfuerzos de control durante más de 60 años. En la actualidad, las regiones centrales restantes endémica *Schistosoma japonicum* se encuentran principalmente en las regiones pantanosas y lacustres a lo largo de la cuenca del río Yangtze.

métodos: Durante el período de 2001 a 2015, una mejora ambiental integrada de las marismas se llevó a cabo mediante la ejecución de proyectos industriales, agrícolas y de desarrollo de recursos en el condado de Yizheng lo largo del río Yangtze. *S. japonicum* la infección en los seres humanos, el ganado y los caracoles se estimó mediante serología, análisis de heces, la técnica de microscopía de eclosión y durante el período de estudio de 15 años para evaluar el efecto de la mejora ambiental integrada en el control y eliminación de la enfermedad.

resultados: Una tasa global de 0,03% *S. japonicum* Se observó infección en los caracoles durante el período de estudio de 15 años, y no se detectaron caracoles infectados desde 2012. La prevalencia global de *S. japonicum* infección fue de 0,87% en los seres humanos durante el período de estudio, y no se encontró infección en humanos desde 2012. Además, sólo 13 bovinos fueron identificados con *S. japonicum* infección en 2003 durante el período de estudio de 15 años, y desde 2004, no se encontró la infección en el ganado.

Conclusión: Los resultados del presente estudio demuestran que la aplicación de proyectos industriales, agrícolas y de desarrollo de los recursos hídricos, no sólo altera los hábitats de caracoles en las regiones pantanosas, y promueve el desarrollo económico local, que parece una estrategia de ganar-ganar para bloquear la transmisión de *S. japonicum* y acelerar el desarrollo socioeconómico a lo largo del río Yangtze.

Translated from English version into Spanish by MKaszczynice, through

TRANSLATORS
WITHOUT BORDERS
